# Normal Ovariotomy—Case

**Published:** 1872-09

**Authors:** Robert Battey

**Affiliations:** Rome, Georgia


ATLANTA

### Medical and Surgical Journal.

Vol. X.—SEPTEMBER, 1872.—No. 6.

---

### ORIGINAL COMMUNICATIONS.

---

### NORMAL OVARIOTOMY—CASE.

BY ROBERT BATTEY, M.D., ROME, GEORGIA.

The surgical operation which I am about to introduce for the first time to the profession, although better entitled to the distinctive designation of *ovariotomy* than the one now generally known and accepted as such, differs so widely in its nature and objects as to merit a new and distinguishing appellation. I have, therefore, taken the liberty of introducing the term *normal ovariotomy*, and of defining it to be *an operation for the removal of the normal human ovaries, with a view to establish at once the "change of life," for the effectual remedy of certain otherwise incurable maladies.* The inception of the ideas that form the groundwork upon which this operation is based is so intimately connected with a case under my charge more than six years ago, that I may be pardoned the digression while I briefly relate it from my note-books.

Case.—Miss Mary ——, aged twenty-one, asked my counsel in October, 1865, with complaint of violent perturbations of her nervous and vascular system, dependent upon amenorrhœa. She was in the bloom of her early womanhood—gifted with charms beyond the lot of the majority of her sex—to all outward appearances perfect in her physical development—her mammæ and external genitalia fitting to her sex and her age. She was gentle and womanly in her manners and sentiments; she enjoyed the society and attention of gentlemen; she had her loves and her estrangements, as other girls of her age. In fact, she was unconscious of anything which marked a distinction between herself and other young women, save only in the lack of that distinctive mark of perfect womanhood—the normal menses. The menstrual molimen was regular and vigorous, and had been for the space of five years, but there had been no issue of blood.

A careful exploration of the pelvis revealed a hymen intact—a vagina normal in its length and breadth and anatomical structure. At the apex of the vagina was a small, firm, fleshy nodule, like a rounded button. There was no appearance of an os uteri, nor of a uterus, except the rudimentary nodule mentioned. The finger in the rectum and catheter in the bladder revealed nothing but the intervening rectal and vesical walls. There were symptoms of endocarditis, with hypertrophy of the heart. Her periodical sufferings were extreme. She, like "the woman who had an issue of blood, had suffered many things of many physicians, and was nothing better, but rather grew worse." She looked imploringly to me for help; what could I do?

There was reason to believe that the cardiac malady in this case had its origin in the amenorrhœa. Nature had provided ovaries performing their natural function in full vigor, but no womb to respond by the proper issue of the menses. I said to myself, if she could be relieved of her ovaries, the balance would at once be restored; the menstrual molimen would cease; the violent strain upon the heart would be at an end; there might be hope for her.

I searched, but searched in vain, for an authority—for a precedent. How dare I to make one! Days, weeks, months, went by—my poor suffering patient calling constantly, but calling in vain, for relief, until finally death, the stern arbiter of all human destiny, answered her call. Inwardly I resolved that another such case should not perish in my keeping without my reaching out a friendly hand in the hope of rescue.

CASE.—Miss Julia ——, aged twenty-three, came under my professional care in July, 1865—from which time to the present I have been charged with her health. She was a bashful, sensitive, retiring girl—more or less an invalid from her childhood, never robust. The menstrual molimen first made its appearance at the age of sixteen, but without the menstrual flux. Twice only had she anything which could be properly termed a normal catamenial flow during the seven years prior to my charge of her. She received at my hands, from time to time, such tonic, restorative and so-called emmenagogue medicaments as have been, and are, of repute in similar cases. At times she increased in flesh and strength, and then again would become much worn down by her periodical efforts at menstruation.

It would be both tedious and profitless to enter into the details of this long and varied treatment. Suffice it to say that little and only temporary advantage was gained by it all, though diligently persevered in for months and years.

In the summer of 1868, all hope of relief by medication having been abandoned, and the wretched state of her general health precluding all idea of marriage, a vaginal examination was instituted, and a diagnosis of chronic corporeal endometritis made. She was subjected to local treatment by the nitrate of silver pencil during the summer and fall seasons, with the result of somewhat improved general health. She now had, with her menstrual efforts, hemorrhages from the stomach, which somewhat relieved the severity of her attacks, and upon one occasion, an apparently natural catamenial flow for two days, which followed a cauterization, just as the menstrual molimen began to show itself. This encouraging sign, however, could not be elicited again.

During the years 1869 and '70, matters grew worse. She had copious and debilitating hemorrhages from the stomach and from the rectum. On three occasions, there was retro-uterine hæmatocele, followed by abscess. She had repeated attacks of acute rheumatism; and more than this, a chronic cough set in, and continued for many months, with characteristic signs of some tubercular deposition in the lungs. Her general health was wretched, and her prospects for improvement were gloomy indeed.

During this period, the thought often recurred to me, if I could but divest her of the source of her menstrual molimen—viz., the ovaries—there would be hope for her. It did not seem that her life could be much prolonged, and her feeble state forbade the idea of risking my own repute, and the reputation of an operation which, though bold and without a precedent, I felt must some day do valuable service in the abridgment of human suffering and the prolonging of human life.

In May, 1871, the intra-uterine treatment was resumed, with the aid of sponge tents, and carried on regularly, and with perseverance, through the summer and autumn, until December. The general health was apparently improved by it; the uterus became to all appearances healthy, but there was no show of the catamenia. She passed the winter without cough; suffered two or three lighter attacks of rheumatism; had her usual paroxysms of amenorrhœa at intervals of about six weeks, with the customary hemorrhages—sometimes gastric, sometimes rectal. In March, 1872, there came another pelvic abscess, discharging per rectum.

Her general condition was now so far improved as to lead me to entertain favorably the idea of a *normal ovariotomy* for her radical cure. For the first time, I suggested the thought to her, and explained fully the nature and character of the operation—its entire novelty, its risks, its probable results. It was presented nakedly, as the creature of my own thought, unsupported by any experience of my own or of any other person—wholly unsupported by the *opinion* even of any living soul but myself.

To my poor suffering and despairing patient, it was as a glimmering flash of light across the dark and portentous sky which had so long overshadowed her life. She seized hold of it with avidity, as doth the shipwrecked mariner a floating fragment of spar. She declared with emphasis that "her life was but a living death;" that to her the bare hope of cure—full and final cure—was of infinitely greater value than the realization of life coupled with her sufferings.

From this time forth, both the patient and her friends were impatient of delay, and importuning for the operation. I demanded the free and full consent of all her relatives; it was promptly given. I asked time to consult a few surgical friends of authority in the land; it was grudgingly granted. There was at no time any persuasion, any urging; there was room for none. The patient a refined, intelligent, educated woman, moving in our best society, I could not urge to the risk of her speedy death, and the certain sacrifice of her sexual character. Nothing but her hopeless extremity could render the operation at all inviting, and of this extremity she alone could be the judge.

Preliminary consultations having been arranged, the operation was appointed for Saturday, 17th August, in order to allow time for her to recover as much as possible from her last attack, the next one being expected in due course about the 24th. On Thursday, the 15th, she informed me that she was experiencing already the prodrome of her coming paroxysm. The question of delay came up; she urged various considerations against it, touching her own personal convenience and the convenience of the family, as well as her own disappointment. I had fixed the day a little in advance, as I supposed, of the usual attack, fully believing that I should put a stop at once and forever to all menstrual impulse, and thus ward off the paroxysm. There seemed good reason to believe, that even though ovulation should be actually transpiring at the time of the operation, if the nervous and vascular disturbances were not already fully set up in the general system, it would thereby be effectually forestalled. It was, therefore, determined to purge the patient well—to enjoin rest, and, if need be, resort to opiates for tranquility, rather than recall the appointment. This determination was further strengthened by the consideration, that another paroxysm would add to her debility, and require additional time for recuperation, with the risk that her then condition might not be so favorable as at present.

OPERATION.

*First Day.*—Saturday, 17th August, 1872, at noon, the operation was done, with the assistance of Drs. G. W. Holmes, W. D. Hoyt, and J. B. S. Holmes, to whom I am under special obligations for their counsel and hearty coöperation.

The patient was chloroformed by Dr. Hoyt, with some difficulty, and owing to her insusceptibility, the anæsthesia compelled a frequent halting and delay. The incision in the median line was begun an inch above the crown of the pubis, and extended upward for the space of three inches. Numerous small vessels sprung, and were temporarily ligatured to save time. There was a full inch of subcutaneous fat. The bellies of the rectus muscles were fully developed, and so closely applied to each other as to form apparently one mass, in which the thin membranous septum marking the linea alba was with difficulty made out; the muscular fasciculi were separated with the finger, and the peritoneum opened upon a director.

The pelvis was now explored with two fingers of my left hand. The uterus, in respect to its size, position and texture, seemed to be perfectly healthy; the ovaries were likewise healthy. There were no abnormal adhesions of the pelvic organs. To the right of and behind Douglas's fossa, was some fibrinous deposit, probably the remains of the abscesses in that region, which had discharged by the rectum and vagina. Notwithstanding the purgatives previously ordered, the sigmoid flexure of the colon was found to be distended with healthy fæces.

The right ovary was quickly brought to the light between my two fingers, and examined. Upon its upper and posterior surface, was distinctly marked *a freshly-ruptured Graafian vesicle*, with a small drop of yet liquid blood exuding from the point of rupture. It seemed as though the ovum had but just escaped. An attempt was made to enucleate the ovary from its peritoneal investment, in order that the ovaric artery might be separately ligatured. This was readily effected in the larger and outer portion of the organ; but elsewhere, the blending of tissues between the peritoneum and tunica albuginea was so intimate as to lead me to abandon the purpose, and the base of attachment to the broad ligament was then transfixed at the centre with a needle carrying a doubled silk, and tied in two equal halves.

The left ovary was now searched for, and seized over the ramus of the pubis, at the left inguinal ring. Upon its surface, it presented likewise the marks of a recently-ruptured Graafian vesicle, with a spot of darker-colored and coagulated blood in the point of rupture. It appeared probable that two, possibly three, days had elapsed since the escape of this ovum, but certainly not five weeks. The pedicle of the left ovary was ligatured by silk in two halves, in like manner, with the other. The ligatured pedicles were held in view sufficiently long to give ample assurance that there could be no possible hemorrhage from their vessels. The ovaries having been previously cut away with scissors, all the ligatures were cut short, and the pedicles allowed to drop back into the pelvis. There was a little oozing of serum stained with blood, which was carefully sponged out, and the walls of the abdomen were now closed by three deep silver wire sutures, including the peritoneum, and secured by large-sized buckshot, perforated through the centre, strung upon the wires, and compressed so as to hug the wires securely. Eight superficial sutures, likewise of silver wire, completed the closure.

The patient was put to bed about 1 o'clock; soon recovered from the chloroform, and complained of great pain in the sacrum, and somewhat, also, of the lower abdomen. The pulse was good, and the general condition very satisfactory. A little brandy was given, and a grain and a half of sulphate morphine per orem.

3 P.M.—Drew four ounces urine by the catheter, and administered per anum one drachms tincture opium. 4 P.M.—Slept a little. 4½ P.M.—Still complains of pain, and had one drachm tincture opium per anum. 6 P.M.—Pulse seventy-two; complains of pain, and had one and a half drachms tincture opium per anum. 7 P.M.—Pulse ninety; drew eight ounces urine. 9 P.M.—Pulse eighty-four; still complains of pain; vomited a greenish water, and had one and a half drachms tincture opium per anum; abdomen rubbed with oil turpentine. 10 P.M.—Drew four ounces clear urine; still complains of pain; had one grain morphine per anum.

*Second Day.*—1 A.M.—Pulse one hundred; vomited; drew four ounces urine; had a grain of morphia per anum. 3½ A.M.—Pulse eighty-eight; vomited, and there was occasional vomiting until daylight. 7 A.M.—Pulse eighty-eight; has slept most of the time since 10½ P.M.; she is free of pain—complains only of soreness, nausea, and a little headache; drew eight ounces urine. 8½ A.M.—Had one grain morphia per anum. 12½ P.M.—Pulse ninety-six; but little heat of skin; drew ten ounces urine; rubbed oil turpentine. 1 P.M.—Had one grain morphia per anum; no vomiting since 5 A.M.; takes a little lime water and milk, with ice, as she wants it. 4 P.M.—Pulse one hundred; skin warm and dry; no vomiting. 5 P.M.—Drew eight ounces urine. 7 P.M.—Pulse ninety-six; still no vomiting; drew two ounces urine; gave one grain morphia per anum; applied turpentine to abdomen. 10 P.M.—Pulse one hundred and twelve; sleeping. 11 P.M.—Pulse one hundred; drew six ounces urine; three-fourths of a grain of morphia per anum; takes lime water and milk; slept well since 8 P.M.

*Third Day.*—2 A.M.—Pulse one hundred; drew three ounces urine; had three-fourths of a grain of morphia per anum; turpentine to abdomen, which is a little distended; sleeping well. 3 A.M.--Half grain morphia per anum. 6 A.M.—Pulse one hundred; drew six ounces urine; one grain morphia per anum; turpentine to abdomen; takes lime water and milk. 11 A.M.—Had half grain morphia per anum. 12 M.—Pulse one hundred and eight; drew eight ounces urine; turpentine to abdomen. 1 P.M.—Pulse one hundred and eight; skin soft—in good, warm perspiration; quite drowsy, but wakes at intervals. 3 P.M.—Pulse one hundred and eight; skin moist; had half grain morphia per anum. 5 P.M.—Had half grain morphia per anum. 6 P.M.—Pulse one hundred and four; drew ten ounces urine; turpentine to abdomen; cloth moistened with diluted carbolic acid (1 to to 24) on the wound. 9 P.M.– Pulse one hundred and eight; had one grain morphia per anum. 11 P.M.—Pulse one hundred and eight; drew three ounces urine.

*Fourth Day.*—3 A.M.—Pulse one hundred and twenty; drew six ounces high-colored urine; had one grain morphia per anum; there is more heat of skin, upper portion body and anus in moist state, abdomen and limbs hot and dry; no pain; lies upon her side, and has slept quietly since 11 P.M. 7 A.M.—Pulse one hundred and eight; skin moist; three ounces urine; one grain morphia per anum. 8 A.M.—Turpentine to abdomen. 11 A.M. Pulse one hundred; drowsy; skin moist and natural; drew four ounces urine. 1 P.M.—Pulse ninety-six; sleeping soundly; good, warm perspiration; abdominal tenderness much lessened to-day; she moves herself with comparative ease in bed. 2 P.M.—Pulse ninety-six; two ounces urine; there is some sanguineous discharge from the vagina, and a little exudation of blood into the bladder. 6 P.M.—Half grain morphia per anum. 7 P.M.—Pulse ninety-six; four ounces urine; skin moist, and there is less heat than last night; renewed turpentine and carbolic acid lotion; the edges of the wound, for an inch on either side, still present a red, rather erysipelatous blush, but not so decided as last night. 8 P.M.—Half grain morphia per anum; vaginal flow increasing. 9½ P.M.—Half grain morphia per anum. 11 P.M.—Pulse one hundred; skin moist; three ounces urine. 11½ P.M.—Half grain morphia per anum.

*Fifth Day.*—3 A.M.—Pulse one hundred and four; sleeps well; three ounces urine. 4½ A.M.—Half grain morphia per anum. 7 A.M.—Pulse ninety-six; four ounces urine. 9 A.M.—Pulse ninety-two; moist skin; resting well. 12 M.—Pulse one hundred and eight; skin hot; four ounces urine; half grain morphia per anum; turpentine and carbolic lotion to abdomen; in half hour, another half grain morphia per anum. 3½ P.M.—Pulse one hundred and four; skin hot and dry; two ounces urine; half grain morphia per anum. 5½ P.M.—Pulse one hundred and four; skin moist and cooler; half grain morphia per anum; three ounces urine. 8 P.M.—Pulse one hundred and twenty; talking in her sleep; more restless for some hours. 9 P.M.—Pulse one hundred and eight; abdomen distended somewhat; erysipelatous blush continues; applied warm poultice. 9½ P.M.—Two ounces urine; half grain morphia per anum.

11½ P.M.—Pulse ninety-six; sleeping soundly; in free perspiration.

*Sixth Day.*—12½ A.M.—Pulse one hundred and thirty; skin rather cold; two ounces urine; half grain morphia per anum; says she "is a great deal better, but knows, by our looks, that we don't think so;" talks rather nervously, excitedly. 3½ A.M. Pulse one hundred and eight, softer; skin warmer; gentle perspiration; four ounces urine; half grain morphia per anum; the mind is clear; she seems very comfortable, is free of pain; bears the touch upon the abdomen greatly better; abdomen is softer, and less swollen; turns herself in bed with much more ease; the nurses see these changes in her suddenly marked—their portent we shall see. 7½ A.M.—Pulse one hundred, good volume; she seems brighter, and more natural; complains of a *sweet taste* constantly in her mouth; four ounces urine; half grain morphia per anum. 9½ A.M.—Pulse one hundred; a little drowsy; says she is more comfortable than at any time since the operation; she took a few mouthfuls of toast at breakfast; drinks iced milk freely; washed the wound and renewed poultice, which is sprinkled with carbolic lotion; wound discharging a little good-looking pus at the lower angle; removed one superficial suture. 11 A.M.—Pulse one hundred; four ounces urine; skin natural; perspiring; had half grain morphia per anum. 2 P.M.—Pulse one hundred and four; four ounces urine; there is a well-marked squint in the left eye, and ptosis of the lid; renewed the poultice to abdomen. 3 P.M.—Pulse one hundred and eight; had half grain morphia per anum. 5 P.M.—Pulse one hundred and eight; two ounces urine. 6 P.M. Had half grain morphia per anum. 9 P.M.—Pulse one hundred and four; had half grain morphia per anum; renewed poultice. 10 P.M.—Five ounces urine. 12 P.M.—Half grain morphia per anum.

*Seventh Day.*—2 A.M.—Drew three ounces urine; had half grain morphia per anum. 7 A.M.—Pulse ninety-six; four ounces urine; she feels rather languid this morning—perhaps by reason of the diminished morphia (?); wound is discharging pus more freely. 11 A.M.—Pulse ninety-six; comfortable; perspiring; eats a mellow pear; renewed poultice, and removed three more of the superficial sutures; the erysipelatous redness is fading out; the edges are for the most part united; a small sinus at the lower angle is discharging a little pus, and a deeper sinus at the upper angle discharges, much more freely, a yellow, rather thin and ill-smelling pus; dress with carbolic lotion on the poultice; she has had no morphine since 2 A.M., and is quite easy and cheerful. 1 P.M.—Pulse ninety-six; four ounces urine; half grain morphia per anum; stop poultice, and substitute carbolic lotion on cloths; changed her clothing. 4 P.M.—Pulse ninety-six; two ounces urine; dressed wound; pus is discharging freely; passed a sound deep down into the abdominal wall, but does not enter the cavity. 6 P.M.—Pulse ninety-six; skin moist; temperature natural; renewed carbolic lotion, and gave half grain morphia per anum. 8 P.M.—Pulse nine-six; four ounces urine; vomited a piece of the pear eaten; renewed carbolic lotion; gave half grain morphia per anum. 11 P.M.—Pulse one hundred; two ounces urine; renewed carbolic dressing; probe now enters the peritoneal cavity, and two ounces of ill-smelling pus escaped; gave half grain morphia per anum. 12 P.M.—Had half grain morphia per anum.

*Eighth Day.*—1 A.M.—Removed another superficial suture, and inserted a compressed sponge tent into the sinus, to secure free exit for pus. 3 A.M.—Pulse one hundred; four ounces urine; gave half grain morphia per anum; removed the sponge tent; it has opened up the sinus admirably, and pus wells up freely from the cavity now—a good, thick, laudable pus; the odor is not so offensive; the sanguineous discharge from the vagina has returned again. 7 A.M.—Pulse eighty; four ounces urine, with sediment; removed three more superficial sutures, and renewed carbolic dressing; she feels more comfortable this morning—moves herself with ease; there seems to be some hardness in the right iliac fossa, which is dull on percussion, and yields pus when the sinus is pressed upon; elsewhere the abdomen is soft and resonant; the sinus is freely open, but not much pus is discharged; injected the cavity with one ounce of very weak carbolic lotion, which came back tinged with pus. 12 M.—Pulse eighty-four; four ounces urine; half grain morphia per anum; vomited milk-curd and bile; stomach a little disordered from the pear on yesterday. 4 P.M.—Pulse eighty-four; slight vomiting; half grain morphia per anum; takes lime water and milk more freely. 5½ P.M.—Threw into the abdomen three ounces carbolic water, which returned promptly, with some pus. 6½ P.M.—Pulse eighty; six ounces urine; still some nausea; the tongue is red and fissured, which is usual with her in her attacks. 9 P.M.—Had three-fourths of a grain of morphia per anum. 10 P.M.—Pulse eighty-four; skin warm, and rather dry; not much influence of morphia. 11 P.M.—Pulse eighty-four; four ounces urine; dressed abdomen; there is but little pus; the bad odor is nearly gone.

*Ninth Day.*—1 A.M.—Pulse eighty-four; has not rested well; gave one grain morphia per anum. 4 A.M.—Pulse eighty-four; five ounces urine; dressed wound; moderate discharge of pus, with but little odor; she has slept well since 1 A.M.; skin natural, no sweat; stomach quiet. 8 A.M.—Pulse eighty-four; five ounces urine, clear; skin is dry, and a little too warm; very little pus; bad odor entirely gone; wound granulating; says she is better than at any time since the operation. 9 A.M.—Pulse eighty; half grain morphia per anum. 2 P.M.—Pulse eighty-eight; skin warm and dry; eight ounces urine, clear and bright color; dressed wound; very little pus; abdomen is now flat and natural. 5½ P.M.—Pulse eighty-eight; skin moist; gave half grain morphia per anum; has had a very comfortable day. 8 P.M.—Four ounces urine; dressed wound. 10 P.M.—Pulse eighty-eight; one grain morphia per anum. 12 P.M.—Pulse eighty-eight; three ounces urine; not resting well—no pain, but restless.

*Tenth Day.*—4½ A.M.—Pulse one hundred; three ounces urine; one grain morphia per anum; she has not rested quite so well; wound is gaping a little by yielding of tissues from the deep sutures. 7 A.M.—Pulse ninety-six; comfortable; has slept. 10 A.M.—Pulse ninety-six; four ounces urine; dressed the wound; tightened the deep sutures by putting on a fresh ball below the old one—a slit sawed into the centre of the ball and compressed upon the wire like a boy's "sinker" on a fish-line; drew the edges well together with three long adhesive strips; very little pus; no bad smell; administered an enema, half pint warm soap-suds, which was allowed to remain. 12 M. Pulse ninety-six; resting comfortably; enema has not returned. 2 P.M.—Pulse ninety-two; two ounces urine; resting quietly. 3 P.M.—She has had a nervous chill, lasting five to eight minutes; extremities congested and warm; gave one grain of morphia, two drachms brandy per orem; reaction soon came on, with pulse of one hundred and twenty; hot, moist skin; complained of urine—drew one ounce; there is tumefaction and great tenderness of the urethra. 4P.M.—Pulse one hundred and twelve; skin moist, and cooler. 4½ P.M.—Enema repeated, and the bowels moved. 5½ P.M.—Gave one grain morphia per anum. 6½ P.M.—Pulse ninety-six; free sweating, dressed the wound; two of the balls have ulcerated through the skin, and are buried deep down in the abdominal walls; they were extracted with some difficulty, and all the deep sutures removed; wound supported by adhesive strips; there are now three sinuses for pus extending down into the cavity—one at the upper angle, and two where the balls had ulcerated through; injected a little carbolic water at each of the three openings, and washed out a little pus; the carbolic lotion tends to loosen the strips; substituted simple cerate dressing. 9 P.M.—Pulse eighty-eight; one grain morphia per anum; she is drenched with perspiration, and complains of restlessness. 11½ P.M.—Eight ounces urine; skin is warmer, and less perspiration.

*Eleventh Day.*—1 A.M.—Gave half grain morphia per anum. 3 A.M.—Pulse eighty; two ounces urine; skin warm, and drying off; took five drops tincture iron in ice water. 5 A.M.—Half grain morphia per anum. 6½ A.M.—Pulse eighty; has just awakened from a long and quiet nap; the skin is natural, the tongue moist, the countenance serene; says she feels much better; the night has been much more comfortable than the previous one. 9½ A.M.—Pulse eighty-eight; skin natural; feels more comfortable than at any time since the operation; drew off four ounces urine, high-colored; gave half grain morphia, and seven drops tincture iron in ice water, per orem, to allow the rectum to regain its muscular tone; dressed wound—it looks healthy; washed out a little pus with syringe, and strapped closely. 1 P.M.—Pulse ninety-two; three ounces urine; dressed wound; very little pus; gave half grain morphia and ten drops tincture iron per orem. 4½ P.M.—Pulse ninety-two; two ounces urine; dressed wound; expresses herself as "feeling very comfortable." 6 P.M.—Pulse one hundred; skin warm and dry; sanguineous vaginal discharge again; gave half grain morphia and ten drops tincture iron. 8 P.M.—Pulse one hundred; sleeping. 9 P.M.—Six ounces urine; gave half grain morphia and ten drops tincture iron per orem.

*Twelfth Day.*—12½ A.M.—Pulse one hundred; three ounces urine; half grain morphia and ten drops tincture iron per orem. 3½ A.M.—Pulse one hundred and twenty; restless; gave half grain morphia and ten drops tincture iron. 7 A.M.—Pulse ninety-six; dressed wound; a mere stain of pus; granulating rather feebly; administered enema of soap and water, which brought away a good, large, pasty evacuation, quite healthy in character, and showing that the purgatives had not properly evacuated the bowels prior to the operation; in about fifteen minutes, had another large fæcal evacuation; can not pass urine voluntarily, yet drew, with catheter, eight ounces. 9 A.M—Pulse ninety-six; skin natural; had half grain morphia and ten drops tincture iron. 1½ P.M.—Pulse eighty-four; skin cool and soft. 3 P.M.—Pulse eighty-eight; perspiration. 5 P.M.—Pulse eighty-eight; skin natural; drew eight ounces urine, very red and turbid; dressed wound; straps are holding well; mere stain of pus; everythings looks very healthy; gave half grain morphia and ten drops tincture iron per orem. 9 P.M.—Pulse ninety-six; four ounces urine, quite red; gave half grain morphia and ten drops tincture iron.

*Thirteenth Day.*—12½ A.M.—Pulse one hundred and eight; skin hot and dry; four ounces urine, more natural; half grain morphia and ten drops tincture iron per orem. 2 A.M.—Pulse one hundred; sleeping. 4½ A.M.—Had half grain morphia and ten drops tincture iron per orem. 7 A.M.—Pulse eighty-four; skin gently moist; drew eight ounces urine—pretty red, not muddy. 11 A.M.—Had half grain morphia and ten drops tincture iron per orem. 12 M.—Pulse eighty-four; gentle perspiration; drew five ounces clear, normal urine. 5 P.M.—Pulse eighty; drew five ounces urine; dressed wound; granulating; no pus; had half grain morphia and ten drops tincture iron.

*Fourteenth Day.*—2 A.M.—Had half grain morphia and ten drops tincture iron. 3 A.M.—Pulse eighty; drew five ounces urine. 8 A.M.—Pulse seventy-six; administered a soap-suds enema, which brought another large fæcal evacuation; wound is granulating slowly; drew six ounces clear urine. 9½ A.M.—Had half grain morphia and ten drops iron. 1 P.M.—Pulse eighty; very comfortable; "feels that she is getting well." 3 P.M.—Pulse seventy-six; urine eight ounces, natural. 6 P.M.—Pulse eighty; had half grain morphia and ten drops tincture iron. 9½ P.M.—Pulse eighty-four; six ounces clear urine; had half grain morphia and ten drops tincture iron.

*Fifteenth Day.*—1½ A.M.—Pulse eighty-eight; drew five ounces urine; had half grain morphia and ten drops tincture iron. 8½ A.M.—Pulse seventy-six; urine evacuated normally for the first time since the operation; enema given—had another large fæcal movement; we are surprised at the intestinal accumulation; had ten drops tincture iron. 2 P.M.—Gave fifteen drops tincture iron. 5½ P.M.—Had half grain morphia and twelve drops tincture iron. 11 P.M.—Had half grain morphia per orem.

*Sixteenth Day.*—1½ A.M.—Half grain morphia per orem. 9 A.M.—Dressed wound; lips are adhering nicely; no pus except a little at the lower angle; urine was passed voluntarily three times during the night; there is considerable scalding during micturition; administered enema—bowels moved, blackened by the iron; she rested quietly all night, but did not sleep very soundly; sat up in a chair to have her bed made this morning. 8½ P.M.—Had one grain morphia per orem.

*Seventeenth Day.*—8 A.M.—Rested well and quietly last night; sat up half an hour this morning to have her bed made. 6 P.M. Has passed a comfortable day. 8½ P.M.—Had one grain morphia per orem.

*Eighteenth Day.*—8 A.M.—Rested quietly all night; slept the first half—wakeful but quiet the latter half; gave enema, but no action; the plasters upon the wound have remained since the sixteenth day—they still hold good; the wound seems to be healing, except at the lower angle. The plasters, which are

English isinglass on heavy bleached linen, are vesicating the skin a little; there is yet scalding in micturition. 6 P.M.—Had a quiet day; bowels moved by enema; ordered ten drops tincture iron three times daily, and one grain morphia at bed-time; she had another action of the bowels during the night, without assistance.

*Nineteenth Day.*—8 A.M.—Rested quietly all night, but did not sleep more than three or four hours; says she is always a night-hawk in health; her condition is very satisfactory every way; sat up three-quarters of an hour. 6 P.M.—Comfortable day; no action of the bowels; took a grain of morphia last night; the urine is still very painful in passing the urethra.

*Twentieth Day.*—8 A.M.—Rested well, and seems bright; sat up three-quarters of an hour; enema—bowels moved; the isinglass plaster, which has remained on now four days, still holds, but it has irritated the wound, and caused portions of the adhesions to melt down, so that it is not doing well; removed it, and applied adhesive strips and simple cerate dressing. 6 P.M. She has suffered a good deal through the day with urethritis.

*Twenty-first Day.*—8 A.M.—Rested badly last night; bowels moved by enema; injected urethra with ten grain solution of nitrate of silver. 6 P.M.—Feels much better; voids urine with much less pain.

*Twenty-second Day.*—8 A.M.—Rested well; feels bright; urine still painful; repeated the injection of nitrate of silver; the wound is dressed twice daily.

*Twenty-third Day.*—Rested well last night; makes complaint of the bladder; there is now a good deal of vesical mucus in the urine; discontinued the nitrate of silver, and ordered injections of solution chlorate potassa three or four times daily, which she says comforts her greatly; at 1 P.M., there was a slight nervous chill, with colicky pains in the bowels; administered enema, and brought away a large amount of hardened fæces, to her great relief.

*Twenty-fourth Day.*—Rested well last night; the bladder is much more comfortable; there is very little vesical mucus discharged; the wound is healed, except the lower angle, which granulates very lazily, on account of the difficulty in keeping the parts approximated; plaster is very poor, and does not adhere well; she is cheerful and bright; gave seidlitz morning and noon, but did not operate; bowels relieved by enema; she has a grain of morphia each night, and ten drops elixir vitriol thrice daily.

*Twenty-fifth Day.*—Had a good night; the pain in micturition is rather more to-day; wound is healing well under the stimulus of occasional touching with nitrate silver; gave seidlitz—no action; enema moved the bowels slightly.

*Twenty-sixth Day.*—Bowels moved by enema; there is much undigested food in the evacuations, and she complains of colicky pains in the bowels; administered a half grain calomel, which moved the bowels twice during the afternoon; the wound is healing and the bladder improving.

*Thirty-first Day.*—Recovery has slowly progressed, retarded only by indigestion, with some colicky pains—due, as I think, to the very free use of morphia during the treatment; the wound is essentially healed, and the pain in micturition is disappearing.

### REMARKS.

The bed-notes in this case were all taken by myself at the bedside, both by day and by night, and they are given as they were taken down, from hour to hour—often with a tired hand and aching brain—without addition or alteration. This fact may explain some imperfections and crudities.

The patient had become habituated to the use of morphia—taken to mitigate her long years of suffering—and her usual dose for the relief of pain had been gradually augmented to a full grain of the sulphate.

On the second day, the weather became intensely hot, and so continued up to the fourteenth day, without a drop of rain to cool the burning earth. The thermometer ranged from 90° to 98° in the shade, and the heat was exceedingly oppressive.

It is my deliberate opinion, that *I have often seen this young lady suffer as much in body and mind, and equally as much in peril to her life, from her amenorrhœal paroxysms, as she has suffered from the surgical procedure to which I have subjected her.* This opinion is fully corroborated by the experience of the patient and by the observation of her friends, in as far as they are capable of judging.

While disclaiming all desire to share any portion of the responsibility of this operation with others, and making no intimation as to individual views privately communicated to me, I desire publicly to express my thanks to Prof. S. D. Gross, of Philadelphia, to Prof. Paul F. Eve, of Nashville, to Prof. Horatio R. Storer, of Boston, and to Prof. W. F. Westmoreland, of Atlanta, for valuable professional courtesies in connection with this case.

As far as my means of information enable me to judge, this operation is *unique* in the annals of surgery—the nearest approximation to it being in the celebrated case of Percival Pott, which is so distinctly stated in his "Chirurgical Works," I may well close my report by its recital:

"A healthy young woman, about twenty-three, was taken into St. Bartholomew's Hospital on account of two small swellings, one in each groin, which for some months had been so painful that she could not do her work as a servant.

"The tumors were perfectly free from inflammation—were soft, unequal in their surface, very movable, and lay just on the outside of the tendons, opening in each of the oblique muscles, through which they seemed to have passed.

"The woman was in full health, large-breasted, stout, and menstruated regularly; had no obstruction to the discharge per anum, nor any complaint but what arose from the uneasiness these tumors gave her when she stooped or moved so as to press them.

"She was the patient of Mr. Nourse. He let her blood and purged her, and took all possible pains to restore the parts through the openings, through which they had clearly passed out. He found all his attempts fruitless, as did Mr. Sainthill and myself, and the woman being incapacitated from getting her bread, and desirous to submit to anything for relief, it was agreed to remove them.

"The skin and membranous adiposa being divided, a fine membranous bag came into view, in which was a body so exactly resembling a human ovarium, that it was impossible to take it for anything else; a ligature was made on it, close to the tendon, and it was cut off. The same operation was done on the other side; and the appearance, both at the time of operating and in the examination of the parts removed, was exactly the same.

"She has enjoyed good health ever since, but is become thinner and more apparently muscular; her breasts, which were large, are gone; nor has she ever menstruated since the operation, which is now some years."

